# Optical Coherence Tomography Angiography Findings in Malignant Hypertensive Retinopathy

**DOI:** 10.18502/jovr.v17i3.11583

**Published:** 2022-08-15

**Authors:** Ahmad Mirshahi, Reza Karkhaneh, Ramak Roohipour, Mohammadbagher Rajabi, Zakieh Vahedian, Fatemeh Bazvand

**Affiliations:** ^1^Eye Research Center, Farabi Eye Hospital, Iran; ^2^Department of Ophthalmology, Morsani College of Medicine, University of South Florida, Tampa, Florida, United States

**Keywords:** Fluorescein Angiography, Malignant Hypertension, Optical Coherence Tomography Angiography, Retinopathy

## Abstract

**Purpose:**

To report the findings of fluorescein angiography (FA) and optical coherence tomography angiography (OCTA) in a patient with malignant hypertensive retinopathy.

**Case Report:**

A 41year-old male was referred to our clinic with sudden visual loss in both of his eyes after an acute rise of blood pressure (200/150 mmHg). Optic disc swelling, flame shape hemorrhages especially around the optic disc, arterial narrowing, vessel tortuosity, cotton wool spots, hard exudate deposition, and multiple deep orange spots (Elschnig spots) were visible in both eyes. In the OCTA, disruption in the normal tapering patterns of the superficial and deep capillary plexuses was observed. Elschnig spots were observed as hypointense spots in the choriocapillaris slab. Leakage of the optic nerve head was seen in the FA.

**Conclusion:**

When compared with the FA, the OCTA can illustrate the ischemic areas and the Elschnig spots with greater detail.

##  INTRODUCTION

The acute rise of blood pressure may influence the retinal and choroidal vessels with multiple clinical features including serous retinal detachment, choroidal ischemia as Elschnig spots, etc.^[1]^ The evaluation of hypertensive chorioretinopathy is possible by using both fluorescein angiography (FA) for retinal circulation and indocyanine green angiography (ICGA) for choroidal circulation.^[1]^ Recently, optical coherence tomography angiography (OCTA) can show all retinal vascular structures in association with choroidal circulation by the detection of erythrocytes movements in vascular structures without any contrast injection.^[2]^

The aim of this study was to evaluate the findings of OCTA in patient with malignant hypertensive retinopathy and comparing it with finding of FA.

##  CASE REPORT

A 41-year-old male who experienced sudden visual loss in both of his eyes five days prior to presentation was referred to our clinic. He had a history of hypertension and experienced an acute rise of blood pressure (200/150 mmHg) five days prior due to forgetting to consume his antihypertensive drugs.

### Ocular examination

**Figure 1:**
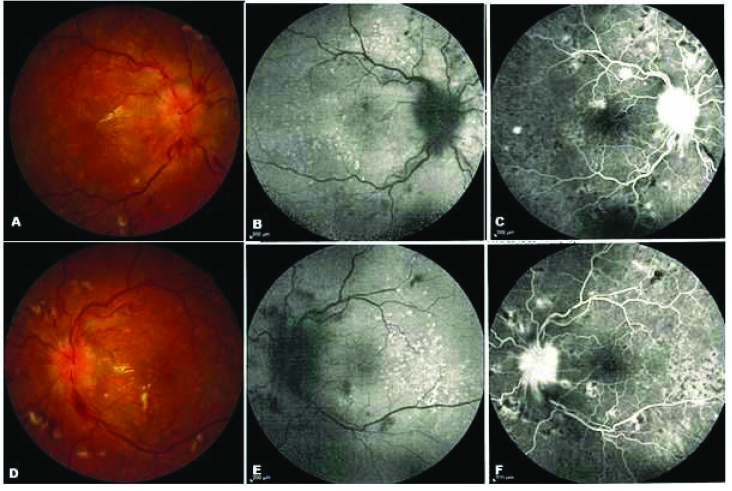
(A & D) Fundus photographs of the right and left eyes show the arterial narrowing, vascular tortuosity, flame-shape hemorrhage, hard exudates, optic disc swelling, and Elschnig spots. (B & E) The fundus autofluorescence illustrates hypoautofluorescence around the optic disc and multiple hyperfluorescence spots compatible with Elschnig spots. (C & F) Fluorescein angiography of both eyes in late phases shows the optic nerve leakage and hypofluorescence spots with hyperfluorescence borders that coincide with Elschnig spots.

The best-corrected visual acuity using the Snellen chart and manifest refraction were, respectively, 20/50 and –1.25 × 50º in the right eye and 20/32 and –0.75 × 110º in the left eye. The ocular examination results including the anterior segment, intraocular pressure, and vitreous examination using slit lamp biomicroscopy and Goldman applanation tonometry were unremarkable in both eyes. The relative afferent pupillary defect was negative. Optic disc swelling, flame shape hemorrhages especially around the optic disc, arterial narrowing, vessel tortuosity, cotton wool spots, hard exudates deposition in the nasal part of the macula together with multiple deep orange spots indicating Elschnig spots were revealed in both eyes in the fundus examination [Figure 1]. Multiple imaging procedures were performed for the patient.

### Spectral Domain Optical Coherence Tomography (SD-OCT) Findings

Irregularity and wrinkling of the retinal surface, slight retinal thickening, subretinal fluid, hyperreflective deposits with posterior shadowing compatible with hard exudates in the inner retinal layers, and some irregularity at the level of retinal pigment epithelial (RPE) cells and the choriocapillaris layer were observed in the SD-OCT of both eyes.

### Fundus Autofluorescence and FA

The hypo-autofluorescence area identified around the optic nerve head (coincident to retinal nerve fiber layer edema), with some scattered hypo-autofluorescence images (coincident to flame shape hemorrhages) and multiple hyper-autofluorescence images particularly in the temporal portion of the macula were all illustrated in both eyes in the fundus autofluorescence imaging [Figure 1]. Optic disc leakage, multiple hypo-fluorescence imaging in the early phase continuing with central hypo-fluorescence and marginal hyper-fluorescence in the late phase coincident with Elschnig spots and some points of blockage in the hemorrhage spots were seen in the FA [Figure 1]. In the FA, only the leakage of the optic nerve head was identified.

**Figure 2:**
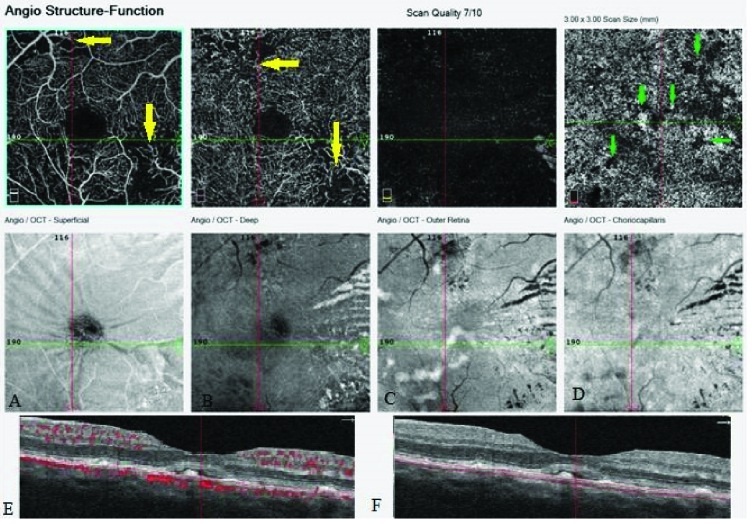
Optical coherence tomography angiography (OCTA) of the right eye. (A) Superficial capillary layer (and the related enface image: hyperreflective spots are seen in the inferonasal of the macula that are compatible with exudates) shows the area of the ischemia and capillary dropout in the inferonasal of the fovea (yellow arrow). (B) Deep capillary layer (and the related enface image: in the location of the deep capillary plexus, exudates as hyperreflective lines that are more prominent in the nasal of the macula in radial pattern) shows the enlargement of the foveal avascular zone and the ischemic area (yellow arrow). (C) The outer retina and the related enface image (the shadowing of exudates are visible as dark lines in outer retina). (D) The choriocapillaris layer (and the related enface image: the shadowing of exudates are also visible as a dark line in the choriocapillaris level and the other hyporeflective spots [Elschnig spots] are also visible in this level) shows hypointense spots (green arrow) that are compatible with Elschnig spots. The subretinal fluid and hyperreflective deposits (exudates) are also visible in B scans.

**Figure 3:**
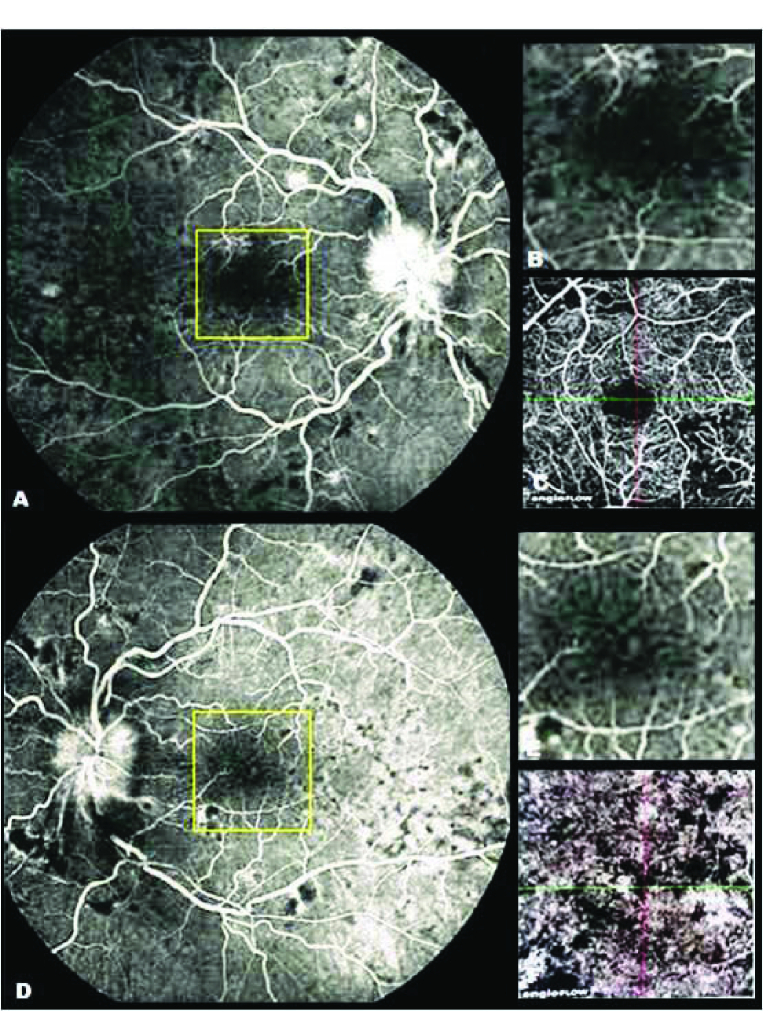
(A) Late phase of fluorescein angiography (FA) of the right eye. (B) The magnified view of the yellow square. (C) OCTA (superficial layer) of the same location of the yellow square; the ischemic areas and the alternation of capillary vessels are obviously observed, however, in the FA these changes are hardly visible. (D) Late phase of the FA of the left eye. (E) The magnified view of the yellow square. (F) OCTA (choriocapillaris layer) of the same location of the yellow square, the ischemic areas and the Elschnig spots are prominently observed, however, in the FA these changes are hardly visible.

### OCTA Findings

In the fovea imaging, the disruption in the normal tapering pattern of the superficial and deep capillary plexuses especially in the inferior nasal and superior temporal of the fovea compatible with the ischemic areas were prominently defined. These findings were marginally revealed in 67 the FA [Figures 2 & 3]. Similar findings with less severity were observed in his left eye [Figure 4]. The 68 Elschnig spots were also revealed at the choriocapillaris level (30 μm below the RPE) in the form of 69 hypo intense spots in both eyes [Figure 2].

##  DISCUSSION 

Hypertensive retinopathy is defined with wide varieties of alterations in retinal vessels comprising diffuse or focal arterial narrowing, vessels tortuosity, hemorrhages and hard exudate deposits due to the hyperpermeability of vessels, and optic disc swelling.^[3,4]^

FA cannot show the majority of the parts of the capillary networks because of light scattering. In spite of that it is the standard method in the assessment of retinal circulation.^[4]^ OCTA is a new amplitude and phase-based imaging technique that can illustrate separately the capillary vessels of the retina together with large vascular structures by sensing the transverse and axial movement of blood cells without any intravenous contrast injection.^[5,6]^

The ischemic areas in both the superficial and deep capillary plexuses in addition to the enlargement of the foveal avascular zone (FAZ) were clearly visible in the OCTA; however, these findings were minimally revealed in the FA on our patient. On the other hand, there were no significant differences between the macular areas of both eyes in the FA, but the best-corrected visual acuity of both eyes showed considerable difference. The amount of ischemic areas and enlargement of the FAZ were obviously larger in both the superficial and deep capillary networks in the right eye as compared to the left eye in the OCTA. It might be approximately proportional with visual acuity. It can be concluded that OCTA may have the prognostic role in the assessment of the visual outcome in vascular diseases.

Consequently, confirmation of this hypothesis requires further studies with larger sample sizes and long-term follow-up.

Focal necrosis in the choroidal arterioles results in ischemia of the overlying choriocapillaris layer and resultant RPE damage that is known as Elschnig spots.^[7,8]^ The existence of Elschnig spots in the choriocapillaris layer were prominently revealed as hypointens lesions in the OCTA in a greater quantity when compared with FA. Elschnig spots are clearly visible in the ICGA as hypofluorescent spots.^[1]^ Kawashima et al^[1]^ reported that more Elschnig spots were visible in the ICGA as compared to the FA. A greater amount of Elshnig spots were revealed and were prominently visible in the OCTA in comparison to the FA. It seems that the OCTA may be of more valuable assistance than the FA in the illustration of hypofluorescent spots in the choriocapillaris layer. Rotsos et al^[7]^ reported a finding of multimodal imaging in two patients with hypertensive chorioretinopathy. They observed hyperreflective lesions in the OCTA of their patients without detection of the focal ischemic area at the choriocapillaris level (Elschnig spots). They proposed that these hyperreflective lesions may be related to fibrin deposits developing on top of the RPE. They mentioned that the lack of displaying of the Elschnig spots on the OCTA might be explained by the resolution of the imaging.^[7]^ The inability of the OCTA in illustrating the Elschnig spots may be partly related to an artifact produced by hyperreflective deposits overlying the RPE. In contrast to their findings, we discovered that OCTA can detect Elschnig spots very well. Consequently, the OCTA is deemed more accurate in detection and better in the visualization of the microvascular structure of the retina and choroid than the FA in diagnosing hypertensive retinopathy.

It may be concluded that the OCTA can illustrate the microvascular changes within the ischemic area in addition to identifying Elschnig spots with greater details than FA in malignant hypertensive retinopathy.

##  Financial Support and Sponsorship

None.

##  Conflicts of Interest

There are no conflicts of interest.
