# Erratum: Optical Coherence Tomography Angiography Findings in Malignant Hypertensive Retinopathy

**DOI:** 10.18502/jovr.v17i4.12351

**Published:** 2022-11-29

**Authors:** Ahmad Mirshahi, Reza Karkhaneh, Ramak Roohipour, Mohammadbagher Rajabi, Zakieh Vahedian, Fatemeh Bazvand

**Affiliations:** ^1^Eye Research Center, Farabi Eye Hospital, Iran; ^2^Department of Ophthalmology, Morsani College of Medicine, University of South Florida, Tampa, Florida, United States

In the article titled “**Optical Coherence Tomography Angiography Findings in Malignant Hypertensive Retinopathy**” published on pages 432–436, Volume 17, Issue3of *Journal of Ophthalmic and Vision Research*,^[1]^ the affiliation of the third author “Ramak Roohipour" is the second affiliation “Department of Ophthalmology, Morsani College of Medicine, University of South Florida, Tampa, Florida, United States". The online version of the article has therefore been updated on October ___, 2022 and can be accessed from ___________.
